# Relationship between Muscular Activity and Postural Control Changes after Proprioceptive Focal Stimulation (Equistasi^®^) in Middle-Moderate Parkinson’s Disease Patients: An Explorative Study

**DOI:** 10.3390/s21020560

**Published:** 2021-01-14

**Authors:** Fabiola Spolaor, Marco Romanato, Guiotto Annamaria, Antonella Peppe, Leila Bakdounes, Duc-Khanh To, Daniele Volpe, Zimi Sawacha

**Affiliations:** 1 Department of Information Engineering, University of Padova, 35131 Padova, Italy; fabiola.spolaor@unipd.it (F.S.); marco.romanato.2@studenti.unipd.it (M.R.); guiotto@dei.unipd.it (G.A.); 2 Istituto di Ricovero e Cura a Carattere Scientifico, Fondazione Santa Lucia, 00179 Roma, Italy; a.peppe@hsantalucia.it; 3 Fresco Parkinson Center, Villa Margherita, S. Stefano, 20010 Vicenza, Italy; leila.bakdounes@casadicuravillamargherita.it; 4 Department of Statistical Sciences, University of Padova, Via C. Battisti 241, I-35121 Padova, Italy; duckhanh.to@unipd.it; 5 Department of Medicine, DIMED, University of Padova, 32128 Padova, Italy

**Keywords:** parkinson’s disease, neurorehabilitation device, wearable focal mechanical vibrations device, surface electromyography, balance assessment, gait analysis

## Abstract

The aim of this study was to investigate the effects of Equistasi^®^, a wearable device, on the relationship between muscular activity and postural control changes in a sample of 25 Parkinson’s disease (PD) subjects. Gait analysis was carried out through a six-cameras stereophotogrammetric system synchronized with two force plates, an eight-channel surface electromyographic system, recording the activity of four muscles bilaterally: Rectus femoris, tibialis anterior (TA), biceps femoris, and gastrocnemius lateralis (GL). The peak of the envelope (PoE) and its occurrence within the gait cycle (position of the peak of the envelope, PPoE) were calculated. Frequency-domain posturographic parameters were extracted while standing still on a force plate in eyes open and closed conditions for 60 s. After the treatment with Equistasi^®^, the mid-low (0.5–0.75) Hz and mid-high (0.75–1 Hz) components associated with the vestibular and somatosensory systems, PoE and PPoE, displayed a shift toward the values registered on the controls. Furthermore, a correlation was found between changes in proprioception (power spectrum frequencies during the Romberg Test) and the activity of GL, BF (PoE), and TA (PPoE). Results of this study could provide a quantitative estimation of the effects of a neurorehabilitation device on the peripheral and central nervous system in PD.

## 1. Introduction

Parkinson’s disease (PD) is a movement disorder that involves many different pathways both of the central and peripheral nervous system. In addition, it is the second most common neurological disease [1,2], affecting 1% of the over 60 years old population [3].

Patients with PD demonstrate balance and gait disorders as alterations in spatio-temporal parameters. Indeed, the gait in these patients is characterized by shuffling steps accompanied by a stooped posture [4,5,6,7]. In advanced PD, other complications may arise such as the so-called axial signs, such as speech, gait and posture, balance disturbances, impaired decision-making, alertness and regulation of emotions, hypomimia [8,9].

As reported in Konczak et al. [10], PD patients are highly affected by kinesthesia. Such a loss of kinesthetic sensitivity is closely linked to the motor deficits. Kinesthesia is commonly defined as the conscious awareness of body or limb position and motion in space and it is based on sensory information on muscle length, contractile speed, muscle tension, and joint position provided by receptors in the muscles, tendons, and joint capsules. The overall information is collectively named proprioception (or muscle sense). Proprioception plays a crucial role in motor function such as reaching and grasping, static balance, and locomotion [11,12,13,14,15]. Therefore, the loss of proprioception in PD patients is responsible for the limitations in their motor behavior such as the lack of precision in performing their movements accompanied by alterations in their postural and spinal reflexes, thus leading to balance and gait alterations [13,15].

Nowadays, the severity of the PD symptoms is clinically evaluated using clinical scales such as the movement disorders society unified-Parkinson’s-disease-rating-scale (MDS-UPDRS) [16]. In particular, the semi-quantitative score, MDS-UPDRS III, provides a macro view of the motor control without characterizing specific patient kinematics [17].

As the basal ganglia are dysfunctional in PD and their role in the motor control of skilled voluntary movements is compromised [18], biomechanics and the motor control of gait in people with PD is a topic of growing interest for researchers and clinicians.

Many studies have reported the presence of specific gait alterations in PD patients both at the level of kinematics and kinetics parameters associated to the risk of falls. However, motor axial signs are often lumped together under the umbrella of balance and gait impairment, while the presence of one symptom does not necessarily occur together with the other one [19,20,21]. First, gait, balance, and postural disabilities often occur together but one feature can be dominant over the others. Moreover, there are documented cases in which these motor symptoms are not always coexistent [22,23]. Therefore, some patients may encounter gait difficulties without balance impairment, whereas others may present with significant postural abnormalities without major gait impairment.

To date, the clinical management of PD has been based on pharmacological and/or surgical therapy. However, none of these approaches succeeded in completely reducing the motor impairment [24].

Recently, Equistasi^®^ (Milan, Italy) has been proposed as a wearable tool to improve balance and gait impairments by means of a proprioceptive stabilizer that emits focal mechanical vibrations, which is able to transform the body temperature into mechanical vibratory energy via the nanotechnology this patch is embedded with. The vibration generates a variation in fiber lengths and has a modulatory effect on the proprioceptive reflex circuit. The focal mechanical vibration applied on the affected muscular areas interacts with the mechanoreceptors, Golgi tendon organs, and the neuromuscular spindles. The stimuli generated by the vibrations transmit the information to the central nervous system [2,25,26,27,28,29]. Stimulated by the vibrations, the higher motor centers improve the proprioceptive information that underlies the motor control. The improvements due to Equistasi^®^ focal vibrations on postural stability and the decreasing of the rate of falls have already been examined, as well as the better outcomes in the clinical scales and spatio-temporal parameters of gait are documented [2,25,26,27,28,29].

Relying on this evidence, the present contribution aims to investigate whether a change in the proprioception induced by this device impacts the peripheral nervous system in terms of balance and surface electromyography (sEMG) signals during gait. Therefore, as a primary outcome, the efficacy of Equistasi^®^ on restoring a more efficient motor control in PD patients was assessed in terms of spatio-temporal parameters, static posture, i.e., Romberg and sEMG during gait. Seeking this aim, a gait analysis study together with a static balance test were carried on before and after the treatment with Equistasi^®^ in a cross-over, double-blinded, double-dummy clinical trial.

## 2. Material and Methods

The study presented here is part of a multicenter, randomized, double-blinded crossover study. Forty patients diagnosed with idiopathic PD were enrolled in four rehabilitation centers in Italy: S. Lucia Foundation in Rome, the Auxologic Institute of Piancavallo Verbania, the Villa Margherita Clinic in Vicenza (Fresco Parkinson Institute), and the Mondino Foundation Neurological Institute of Pavia. Each center obtained approval from the local ethics committee (protocol number CE/PROG 478/15 del 19/11/2015, 58/16, 61/16, 60/16, respectively). The study entitled “Efficacy of Proprioceptive Focal Stimulation (EQUISTASI) on Gait Parameters in Parkinson Italian Multicenter Study” has been registered in ClinicalTrials.gov (no. protocol NCT02641405).

### 2.1. Subjects

Twenty-five patients affected by idiopathic PD were randomly selected within the total sample to undergo surface electromyography and of them 20 (seven females and 13 males) completed the study (demographic data in Table 1). Five of them did not complete the study due to worsening conditions not related to PD, which required immediate treatment outside the clinical study.

All the patients gave a written informed consent. All the PD participants were enrolled based on the following inclusion/exclusion criteria: The Hoen and Yahr (H/Y) scale of 2–3 and MMSE > 24, which were able to walk autonomously and to perform the required tasks, were on a stable treatment regimen for at least 3 months with a disease duration > 5 years. Furthermore, patients had a good response to the anti-Parkinsonian therapy. Exclusion criteria were the presence of significant freezing of gait, severe balance disorders, co-morbidities that prevent safe mobility, severe dysautonomia with hypotension, major depression, dementia, pregnancy, cardiac pace-makers, DBS, poor visual activity, and vestibular dysfunction. In this study, plaquettes of Equistasi^®^ were added to the pharmacological therapy and no rehabilitation training during the study was performed by patients. Equistasi^®^ is a Class I Medical Device with European Certification registered in Italy as Postural Stabilater no. 231535. The fibers of Equistasi^®^ polymer exclusively made of nanotechnological fibers, are very sensitive to the smallest variation in temperature. These fibers release focal vibration transforming thermal energy which they receive from the skin into mechanical energy. Focal vibration starts a few seconds after the application of the device on the patient’s skin [30,31].

Ten healthy subjects were recruited as the control group (C) (age 65.5 ± 7 years, height 1.61 m, weight 74.5 kg, and BMI 28.7 kg/m^2^) among the clinical personnel matched with PD patients in terms of age and BMI. Exclusion criteria were patients older than 80 years of age, cardiovascular, neurological or psychiatric disease, and severe visual or auditory impairments (the reduced visual acuity was accepted if adequately corrected). Patients with other orthopaedic diseases or previous surgery at the lower extremities were also excluded.

### 2.2. Equistasi Device

Equistasi^®^ is a wearable and innovative medical device, approved by the Ministry of Health on 5th August 2020 with the CNN product code number 342,575 and COP product code number 342,577. It is based on vibrational technology: It self-generates focal mechanical vibrations at a non-constant frequency of about 9000 Hz, within the limits imposed by Legislative Decree 81/08.

Equistasi^®^ has characteristics that make it, to date, a highly innovative tool:

- It is wearable: This device is applied as a regular bandage strip during daily motor activities with total independence or during the rehabilitation treatment carried out by a physiotherapist or professional in the sector.
- It is lightweight: This device is 1 × 2 cm in size and has a feather-light weight of only 0.17 g.
- It is multi-applicable: This device can be reused several times and in different areas for different issues, depending on the instructions of the therapist or doctor.
- It will not expire: Other than general wear, this device will never expire.

Equistasi^®^ is composed exclusively of applied nanotechnology fibers and does not contain any pharmacological elements. It provides support for the patient’s rehabilitative therapy.

Focal mechanical vibration applied on the affected muscular areas interacts with the mechanoreceptors, the Golgi tendon organs, and the neuromuscular spindles. The stimuli generated by the vibrations transmit the information to the central nervous system. Stimulated by the vibrations, the higher motor centers improve the proprioceptive information that underlies the motor control. For these reasons, the application of Equistasi^®^ proves to be an innovative and particularly interesting rehabilitative therapy [32].

### 2.3. Randomization and Blindness

A series of random numbers without repetition (from 0 to 300) were created as in Peppe et al. [2]. Each number was alternately associated with a kit containing three active devices or a kit containing three placebo devices. Each pair of kits (one placebo and one active) were put into a box and sent to the researcher. Each single box was associated with a patient, who randomly chose one of the two kits as the first therapy and the other one as the second. In this way, both the patient and the researcher were both blind to the device type.

### 2.4. Study Design

**Figure 1:**
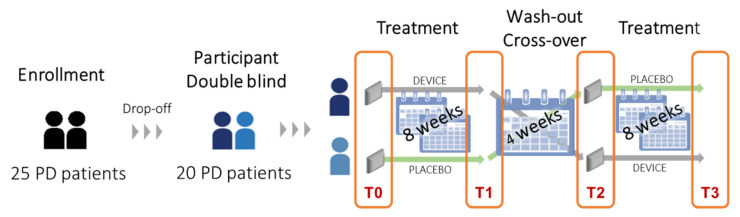
Study design.

After the enrolment, subjects were randomly divided in two groups either to receive an active proprioceptive mechanical stimulation for eight weeks with an active device or to receive a placebo device, in the absence of any other rehabilitative procedure. A 4-week wash-out period has followed the first 8-week treatment period. Then, the patients were switched to the second 8-week period of the other treatment (Figure 1).

According to the US National Library of Medicine [33], a washout period is defined as “*a period of time during a clinical study when a participant is taken off a study drug or other medication in order to eliminate the effects of the treatment*”.

The device was applied on the skin as follows: One over the 7th cervical vertebra and two over each soleus muscle, according to the literature data [25]. The device was worn for 6 days/week during the first week for 1 h/day. During the following 3 weeks, the wearing time was increased by 1 h per week, until 4 h/day on the 4th week. During the following 4 weeks, the wearing time was stable, and the device was worn 4 h/day and 5 days/week. The consistency of the study was monitored by telephone contacts with caregivers and patients. We assessed the outcomes at four time points:

- (T0) baseline before enrolling;
- (T1) second assessment at the end of the first 8-week treatment period;
- (T2) third assessment at the end of the 4-week wash-out period, coinciding with the beginning of the second treatment period;
- (T3) fourth assessment at the end of the second 8-week treatment period.

From here to the end of the manuscript, the data acquired before Equistasi^®^ will be called BE, and the data acquired after Equistasi^®^ will be called AE. Similarly for the placebo, data before and after the placebo will be called BP and AP.

### 2.5. Gait Analysis Protocol

The measures have been acquired at the Human Movement Bioengineering Laboratory (10 m walkway) of the Department of Information Engineering at University of Padova (Italy) by means of a six-cameras stereophotogrammetric system (60–120 Hz of BTS srl) synchronized with an 8-channel sEMG system (1000 Hz, 16 bit A/D resolution, CMRR > 110 Db 50–60 Hz, gain 1000, 500 and 250, respectively with the range of 1.5, 3 and 6 mV, and input impedance >10 GOhm (BTS srl)) and two force platforms (960 Hz, Bertec Corporation, Columbus, OH, USA). A modified version of the IOR-Gait protocol as in [34] was adopted both for anatomical landmarks identification and joint angles calculation. Data collection included static acquisitions for both anatomical calibration and balance assessment. The latter consisted of an instrumented Romberg Test, where the subjects were acquired for 60 s in both eyes open (EO) and eyes closed (EC) conditions, with their arms along the body and the feet 30° apart (assured by a cardboard triangle), while standing still on the force plate [34,35]. For gait analysis, several self-selected speed gait trials were acquired in order to obtain a sufficient number of walking trials where the foot was naturally landing on the force plate, and then detecting the gait cycle. Hence, at least three right and three left foot contacts with the force plate were collected. The assessed variables were represented by the mean from six representative walking trials per subject, selected after applying an intra-class correlation threshold of 0.75 as in Volpe et al., 2017 [28]. The sEMG activity was collected bilaterally on the following lower limb muscles: Rectus femoris (RF), biceps femoris (BF), tibialis anterior (TA), and gastrocnemius lateralis (GL). Sensors were positioned according to Blanc and Dimanico [36] after appropriately cleaning and preparing the skin. Sensors were 24 mm in diameter and positioned 1 cm apart.

## 3. Data Processing

### 3.1. Romberg Test

From the Romberg Test in both EO and EC conditions, the following variables were extracted: Variables which describe the center of pressure displacement over the force platform as ellipse area, sway area, path and velocity, and also variables regarding the frequency of center of pressure oscillation in a spectrum between 0.01 and 5 Hz: 0.03–0.1, 0.1–0.25, 0.25–0.35, 0.35–0.50, 0.50–0.75, 0.75, 1.00, 1.00–3.00, and 3.00 Hz and above [37]. Each one of these frequencies indicates a greater or lower oscillation in the anterior posterior direction (z) and medial lateral direction (x).

Low frequencies (0.03–0.1 Hz) are linked with visual control, and they typically dominate the normal steady and undisturbed posture. The medium-low frequency band (0.1–0.50 Hz) is sensitive to vestibular stress and disturbances. The medium-high frequencies (0.50–1.00 Hz) reflect the somatosensory activity and postural reflexes mediated by the lower extremities. Bursts of high frequencies (over 1 Hz) are often induced by dysfunctions [38,39,40,41,42].

### 3.2. Spatio-Temporal Parameters

In terms of spatio-temporal parameters, the following variables were analyzed:

Stance time in percentage of the gait cycle, stride length (m), gait cycle duration in seconds, gait velocity (m/s), swing time in percentage of the gait cycle, gait cadence (step/min), stride length in percentage of the subject’s height, velocity in percentage of the subject’s height/time (% h/s), and the double support time (s) [43].

### 3.3. sEMG

The recorded sEmg signals of the acquired muscles were processed with MATLAB R2019b (Mathworks Inc., Natick, MA, USA). The raw signals were band pass filtered between 10 and 450 Hz with a 5th order Butterworth filter and full wave rectified. The envelope was computed by low-pass filtering the signals with a 4th order Butterworth filter and a cut off frequency of 5 Hz as in [44]. The right and left muscle activation patterns were analyzed and the following parameters were extracted: Peak of the envelope (PoE) normalized on the mean value during each gait cycle, and its occurrence (PPoE%) within the gait cycle [44]. The sEMG’s envelope mean ± 1 standard deviation of each muscle analyzed are reported and the before and after Equistasi^®^ treatment bands have been overlapped with normative bands in controls.

## 4. Statistical Analysis

Our study is part of a multicenter study, where the duration of both the treatment and washout periods allowed to efficiently detect statistically significant improvements in the cohort of PD patients in terms of UPDRS and spatio-temporal parameters between the different time frames of the study of Peppe et al., 2019 [2]. The Wilcoxon signed rank test was adopted in order to compare those variables characterized by a single sample for each patient, before and after both Equistasi^®^ and placebo treatments. Meanwhile, to compare the same variable between C and PD patients, the Wilcoxon rank sum test was performed. Statistical analysis was carried out using SPSS (version 21.0).

Considering that for sEMG variables repeated measurements were available (six samples were available for each variable at two time points, AE and BE), and in order to assess the treatment’s effect, linear mixed-effect (LME) models were applied to TA PoE, GL PoE, RF PoE, and BF PoE. In particular, the treatment times (BE, AE, BP, and AP) were considered a fixed effect and treated as a nominal factor, the patients were treated as random effects, and the foot factor was treated as nested within patients. In order to guarantee the assumptions of the LME model, we applied the Box-Cox transformation for the LME [45]. After fitting the LME models, we estimated the difference of the sEMG variables means (TA PoE, GL PoE, RF PoE, BF PoE) between the pairs of times (AE–BE, BP–BE, AP–BE, and AP–BP).

To investigate the relationship between the sEMG variables and both the posturographic and the spatio-temporal parameters, we employed a non-parametric correlation test named Kendall’s rank correlation test. In order to perform these tests, for each variable of the sEMG, we considered the average of six replicated measured values (three right-foot and three left-foot) of each patient. The correlation analysis was done using SPSS (ver.21), and a significant correlation is the one with the *p*-value less than 0.05.

## 5. Results

In the following section, results of the analysis were reported. Firstly, results of each assessed variable were hereby presented, followed by the correlation analysis.

### 5.1. Romberg Test

**Figure 2:**
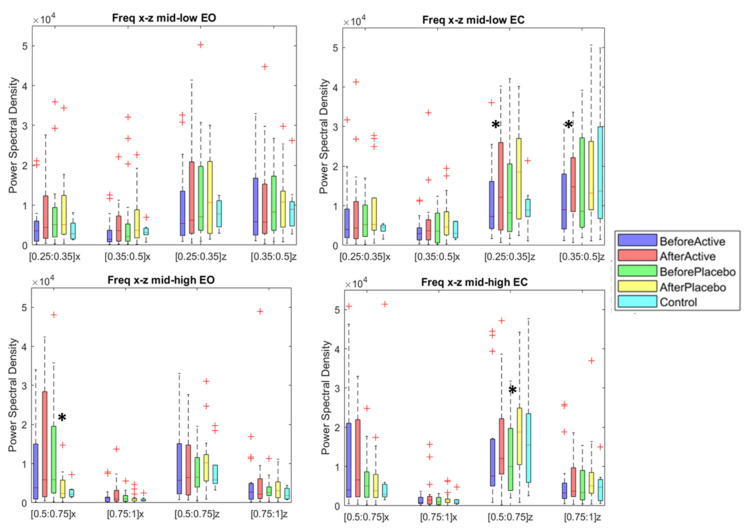
Romberg Test. Differences in the treatments effect and controls accounting for the low and high bands of the postural oscillation. Results are reported in both the medial-lateral component (x) and anterior-posterior component (z). *: Statistically significant difference (*p* < 0.05) between the before-after condition with the Wilcoxon signed rank test (*p* < 0.05). red † indicate the outliers.

All the results listed in the section “Material and Methods” subsection “Romberg Test” were reported in Supplementary Materials. By considering the aim of our study, we focused on the quantitative and qualitative information derived from the center of the pressure spectral analysis. The mid-low (0.1–0.5) Hz and mid-high (0.5–1 Hz) components respectively associated with the vestibular and somatosensory systems were reported in Figure 2, both for EC and divided in the medial-lateral (x) and anterior-posterior (z) components. It can be noticed that after the Equistasi^®^ treatment, the values recorded were closer to the values of the C, and this can be associated with a reduction of the postural efforts and the peripheral vestibular disorders. After the treatment with the Equistasi^®^ device, statistically significant differences were recorded in the mid-low frequencies for the anterior-posterior axes in the EC condition. For the mid-high frequencies, statistically significant differences were reported after the placebo treatment in both conditions and on both components (x, z) (see Figure S2 in the Supplementary Materials).

### 5.2. sEMG

**Figure 3:**
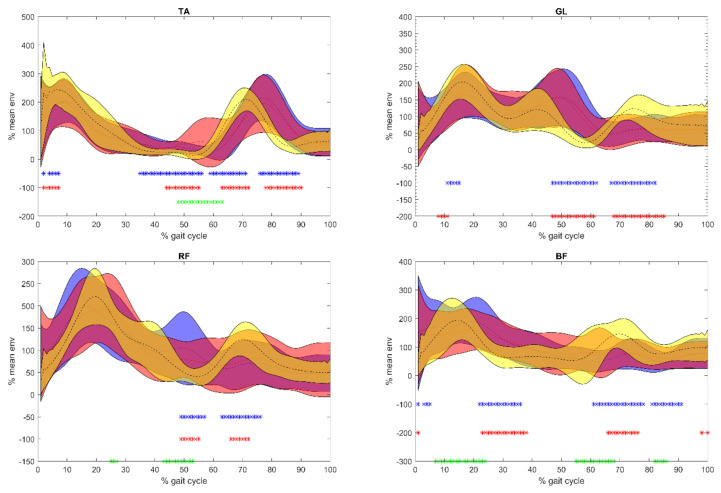
Envelope mean ± 1 standard deviation extracted from the surface electromyographic signal of each muscle analyzed: On the y-axis, the peak of the envelope (PoE in % of mean activation during gait), on the x-axis, the gait cycle (from 0 to 100%). The bottom on the left tibialis anterior (TA) and on the right gastrocnemius lateralis (GL); down on the left rectus femoris (RF) and on the right biceps femoris (BF). In blue PoE before Equistasi^®^, in red after Equistasi^®^, and in yellow normative bands of control subjects. Blue *: Before Equistasi^®^ compared with controls; red *: After Equistasi^®^ compared with controls; green *: Before Equistasi^®^ compared with controls after Equistasi^®^.

**Figure 4:**
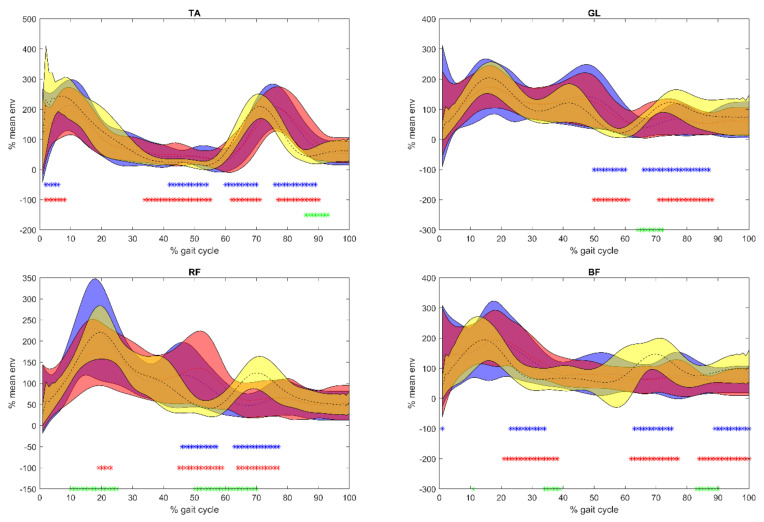
Envelope mean ± 1 standard deviation extracted from the surface electromyographic signal of each muscle analyzed: On the y-axis, the peak of the envelope (PoE in % of mean activation during gait), on the x-axis the gait cycle (from 0 to 100%). The bottom on the left tibialis anterior (TA) and on the right gastrocnemius lateralis (GL); down on the left rectus femoris (RF) and on the right biceps femoris (BF). In blue, PoE before placebo, in red after placebo, and in yellow normative bands of control subjects. Blue *: Before placebo compared with controls; red *: After placebo compared with controls; green *: Before placebo compared with after placebo.

Results of the sEMG analysis were reported in Figure 3 and Figure 4. Before Equistasi^®^ in PD patients, a higher PoE during load acceptance was registered on GL, RF, and BF when compared with the C, and this value decreased after Equistasi^®^ becoming closer to the values registered on the C. Meanwhile, between 25 and 50% of the gait cycle before Equistasi^®^, PD subjects displayed a higher and delayed PoE than C, which in terms of PPoE moved closer to C after the treatment. With the exception of TA, between 50 and 60% of the gait cycle, a shift towards the values observed on the C was registered in terms of both PoE and PPoE, while during the swing phase the lower PoE recorded over all muscles slightly increased after the treatment. The differences observed during the midstance phase of gait suggest a change in the motor control at the level of the thigh muscles after the treatment.

It should be mentioned that TA showed a different behavior with respect to the other muscles: A lower PoE was recorded from the heel strike to the midstance while a higher and delayed PoE was registered from the midstance through the all of the gait cycle before and after Equistasi^®^. An increased activity was also recorded during the push off phase. On the other hand, GL showed a higher and delayed PoE in PD patients with respect to C during the stance phase of the gait cycle accompanied by a lower PoE during the swing phase before the treatment. These differences were partially reduced after the treatment.

Overall, the differences between before and after Equistasi^®^ revealed a change in the motor control after the treatment.

When considering the changes observed after the placebo administration with respect to the ones described after Equistasi^®^: At the level of TA, the increased PoE at push off was not recorded. On the GL, the delay observed at PPoE on the PD group between the end of the stance phase and the swing phase (50–60% of gait cycle) was not reduced. An opposite trend was observed on both the RF and BF, where RF showed a reduced PoE at the loading response and an increased PoE at push off, and at BF, a reduced PoE was detected from 40% until the end of the gait cycle (Figure 3 and Figure 4).

### 5.3. Spatio-Temporal Parameters

The results of the spatio-temporal parameters analysis were reported in Table 2 in terms of the mean and standard deviation. As expected, statistically significant differences were detected between PD and C. As observed for the EMG parameters, differences on the parameters between after Equistasi^®^ and after placebo administration were detected: After the therapy with the active device, subjects denoted a statistically significant increase in stride length accompanied by an increment but not significant of the stride time and velocity. While after the placebo administration, a statistically significant reduction on the stride time accompanied by a reduction, but not significant, in the stride length was observed.

### 5.4. Kendall’s Correlation

All the results of Kendall’s correlation were reported in the Supplementary Materials from Figures S3–S7. According to the aim of our study, only the results on sEMG and both spatio-temporal parameters and medium-high frequencies of the Romberg Test were reported and discussed below.

#### 5.4.1. sEMG and Spatio-Temporal Parameters

Before Equistasi^®^, a negative correlation between PPoE of TA and the stride period was observed. After Equistasi^®^, a negative correlation between PPoE of TA and the stride period was observed. Meanwhile, a positive correlation between the PoE of GL and the stride period was detected.

The main change that occurred after the treatment should be considered the association between the longer stride period and the increased activity of GL in terms of PoE. Before the placebo, a positive correlation was registered between PPoE of TA and both the stance and the double support periods accompanied by a negative correlation with the stride and the swing phases, and the velocity. Furthermore, the PoE of TA showed a negative correlation with the double support period. After the placebo, a positive correlation between PPoE of TA and both the stance and double support periods was detected, accompanied by a negative correlation with the swing period. A positive correlation between PoE of BF and the stride time and a positive correlation between PoE of GL and the stride time were also observed.

#### 5.4.2. sEMG and Romberg Test

Before Equistasi^®^, a negative correlation between TA PoE and 0.75–1 x during EO was observed. After Equistasi^®^, a positive correlation between GL PoE and 0.5–0.75 z during EO, a positive correlation between BF PoE and 0.75–1 x during the EC condition, and a positive correlation between TA PPoE and 0.5–0.75 z in the EC condition were observed.

These results highlighted the association between the changes in proprioception, in terms of power spectrum frequencies recorded during the Romberg Test, and the activity of GL, BF in terms of PoE, and TA in terms of PPoE. Patients who improved their postural control also showed an improvement in the activity of these muscles. Before the placebo, a positive correlation between GL PoE and 0.75–1 x during the EC condition was observed. Meanwhile, after the placebo, a positive correlation between PPoE of BF and 0.75–1 z during OE was observed.

#### 5.4.3. Linear Mixed Model (Treatment Effect on sEMG)

The diagnostic model indicated that the assumptions for the LME models were essentially met under the Box-Cox transformation. In summary, the treatment had an effect on sEMG variables in terms of PoE, but the levels of the effects depend on the type of lower limb muscles. The results were reported in Table 3, where *BE* stands for the mean of sEMG variables at BE; X–BE stands for the different means of sEMG variables between time X and BE; and AP–BP stands for the different means of sEMG variables between time AP and BP.

The significant effects were found in some models with a *p*-value less than 0.05. In summary, we observed:

- For the TA PoE variable, the LME model showed that the average of TA PoE measured in AE and AP were significantly smaller (around 31 and 29 units) than the average obtained in BE.
- For the GL PoE variable, we noticed that the average of GL PoE measured in BP was significantly smaller (around 18 units) than the average obtained in BE, whereas the average of GL PoE measured in AP was significantly greater (around 37 unit) than the one obtained in BP.
- For the BF PoE variable, we observed the same behavior as in the analysis of the GL PoE variable.

## 6. Discussion

This study aimed to evaluate whether a change in the proprioception induced by the wearable proprioceptive stabilizer Equistasi^®^ impacts the peripheral nervous systems in terms of both balance and sEMG of the lower limb during gait. To this end, coupling between changes in the motor control in terms of sEMG and changes in the proprioception measured during the Romberg Test were investigated. The assessment of sEMG signals showed the presence of statistically significant changes in the muscular activity after the Equistasi^®^ treatment, which were different from the ones revealed after the administration of the placebo.

Regarding the spatio-temporal parameters, similar results as in [46], were revealed.

For instance, when considering Kendall’s correlation analysis performed between the sEMG signals and the spatio-temporal parameters, and before and after the treatment with Equistasi^®^, PD patients showed correlations between both the PPoE and PoE and the spatio-temporal parameters [47].

Before the Equistasi^®^ treatment, PD patients with a delayed activation of the TA were more likely to display difficulties in step management in terms of the shorter stride period. Meanwhile, after the Equistasi^®^ treatment, we observed an higher activation of the GL associated with a longer stride period, closer to the control subjects. This could be interpreted as an improvement in gait stabilization: A longer stride period indicates an ability to perform a faster gait. This observation is in accordance with the results reported in a previous study of Peppe et al. [2], where an overall improvement was detected in the spatio-temporal parameters after the treatment with Equistasi^®^.

Furthermore, the treatment effects on sEMG were observed on TA, in which a reduced activity could be interpreted as a more efficient gait strategy. On the other hand, an increased sEMG activity was observed after the placebo in both GL and BF, indicating the recruitment of additional motor units by the central nervous system to accomplish the required task.

Concerning the Romberg Test, previous posturographic studies in PD patients reported contradictory results [48,49]. Differences in a wide range of findings might be due to the use of different assessment methods and data analysis, along with differences in the stage of the disease and severity of motor deficits in the assessed participants. In the present study, we evaluated the static postural control using a force platform and we focused on the information computed from the spectral analysis of the postural oscillation on the medium–high frequencies band (0.50–0.75 Hz and 0.75–1.00 Hz), which are respectively reflective of the somatosensory activity and postural reflexes mediated by the lower extremities.

Indeed, our results showed that after the Equistasi^®^ treatment the values recorded were closer to the values of C, in particular, in the mid-low frequencies for the anterior-posterior axes in the EC condition. This can be associated with a reduction of the postural efforts and the peripheral vestibular disorders [50].

Concerning the associations between sEMG during both the gait and frequency analysis and the Romberg Test, we observed that before the treatment with Equistasi^®^, PD patients who displayed a better proprioception (i.e., lower value of power spectral density) showed a higher PoE at TA during the EO condition. Meanwhile, after the treatment with Equistasi^®^, they showed a lower PoE on GL in the EO condition, accompanied by an earlier PPoE of TA, even with an higher PoE of BF during the EC condition.

These results suggest that in our PD population, after the treatment, the calf muscles play a better role in control stability [51].

As postural control involves neuromuscular coordination and biomechanical interactions, as well as multiple sensory feedback loops, and due to the fact that the stretch reflex arch, contributes to lower-extremity motor responses during both standing and walking [52], the uncoupled evaluation of balance and dynamic muscular activity could be misleading or not informative enough.

Therefore, we evaluated the efficiency of the device for the rehabilitation of the proprioceptive system in PD assessing the interplay between the balance and dynamic muscular activity.

Our results find an agreement with the state of the art reporting that vibration stimuli act as a powerful proprioceptive stimulus that strongly affects motion perception in patients with neurological disorders and, in particular, can cause changes in the motor control patterns of patients with damage at the basal ganglia level [46]. Muscle stimuli applied through the Equistasi^®^ device could help in overcoming the suppression of the thalamus, which plays an important role as a gateway and acts as an integration between the cerebral cortex and subcortical signals, even for a short time [46].

The limitations of the current study should be acknowledged, and in particular, the test adopted for the assessment of proprioception limitation, which is generally carried out in clinics through the H reflex stimuli [27]. In neurophysiology, it is an established concept that muscle tendon vibration determines a change in the discharge frequency of muscle spindle afferents, and it has been demonstrated that the application of a high-frequency tonic vibratory stimulus over a muscle tendon induces the tonic activity of the muscle, which is called the tonic vibration reflex [27]. Based on this assumption in [27], the effects of Equistasi^®^ on the tonic vibration stimulus induced the inhibition of the soleus muscle H-reflex and concluded that Equistasi^®^ has a modulatory effect on proprioceptive reflex circuits.

A further limitation that should be acknowledged is the change that was observed in the spatio-temporal parameters after the placebo intervention, which is different from the change after Equistasi^®^. This finds agreement with Quattrone et al. [53], who also observed a change after the placebo administration in PD.

Future developments should include the increase in sample size, a longer follow up period, and the assessment of the interaction of Equistasi^®^ with the proprioceptive system at both the level of frequency analysis during the Romberg Test and on tonic vibration stimulus-induced inhibition of the soleus muscle H reflex.

In conclusion, this study suggests that focal mechanical vibrations exerted by a wearable postural stabilizer can promote effective motor control strategies in PD patients at both levels: Balance and gait control.

## Figures and Tables

**Table 1:** Demographic data and characteristics of the study participants (n = 20). BMI: Body mass index; LEDD: Levodopa equivalent daily dose; H/Y: Hone and Yare scale.

	Mean	±SD
Age (years)	67.46	10.27
Weight (kg)	77.06	15.86
Height (m)	1.70	0.11
BMI (kg/m^2^)	26.31	3.23
Years of disease	11.88	3.23
UPDRS III Total	36.04	16.6
UPDRS III SCALE ITEM 10	1.55	0.74
UPDRS III SCALE ITEM 11	0.5	1.05
UPDRS III SCALE ITEM 12	1.8	0.8
UPDRS III SCALE ITEM 13	1.45	1.06
LEDD (mg)	757.14	290.88
HY	2.46	0.51
ABC Scale	35.41	12.61
PDQ-39	50.71	24.50
Fall Rate (last month)	0.14	0.47

**Table 2:** Effectiveness of the treatments on spatio-temporal (ST) parameters.

	Active		Placebo		CS
	Before	After	Before	After	
	Mean (±SD)	Mean (±SD)	Mean (±SD)	Mean (±SD)	Mean (±SD)
Stride (m)	0.93(0.22) °	0.96(0.18) *°	0.91(0.17) °	0.91(0.17) °	1.10(0.10)
Time (s)	1.13(0.14)	1.16(0.16)	1.20(0.20) °	1.16(0.18) *	1.00(0.10)
Velocity (m/s)	0.84(0.22) °	0.85(0.20) °	0.78(0.19) °	0.81(0.23) °	1.01(0.10)
Stance (% gait cycle)	63.34(3.18) °	63.10(3.55) °	63.33(3.55)	63.82(3.35) °	61.40(2.90)

°: Statistically significant compared to the C (Wilcoxon rank sum test (*p* < 0.05)). *: Statistically significant between different before and after treatments (Wilcoxon signed rank test (*p* < 0.05)).

**Table 3:** Different means of sEMG variables: TA PoE, GL PoE, RF PoE, BF PoE between the pairs of times (AE–BE, BP–BE, AP–BE, and AP–BP). The results obtained by fitting the linear mixed-effect (LME) model for sEMG variables under the Box-Cox transformation.

		Estimates	Standard Error	95% Confidence Interval	*p*-Value
TA PoE	BE	300.625	11.850	(277.399, 323.851)	<0.001
	AE–BE	−30.729	9.678	(−49.697, −11.762)	<0.001
	BP–BE	−10.688	9.076	(−28.477, 7.102)	0.232
	AP–BE	−29.085	11.178	(−50.993, −7.177)	0.002
	AP–BP	−18.397	10.590	(−39.152, 2.358)	0.082
GL PoE	BE	257.112	11.416	(234.737, 279.487)	<0.001
	AE–BE	5.385	13.193	(−20.474, 31.243)	0.687
	BP–BE	−17.928	6.921	(−31.492, −4.363)	0.007
	AP–BE	19.458	10.586	(−1.291, 40.206)	0.100
	AP–BP	37.385	7.619	(22.453, 52.318)	<0.001
RF PoE	BE	268.189	7.380	(253.724, 282.653)	<0.001
	AE–BE	−23.210	11.335	(−45.427, −0.993)	0.273
	BP–BE	−0.876	11.534	(−23.482, 21.73)	0.939
	AP–BE	6.069	15.010	(−23.349, 35.488)	0.730
	AP–BP	6.945	12.840	(−18.221, 32.111)	0.589
BF PoE	BE	283.075	14.048	(255.541, 310.609)	<0.001
	AE–BE	−20.944	17.604	(−55.446, 13.559)	0.233
	BP–BE	−33.881	14.025	(−61.37, −6.393)	0.020
	AP–BE	9.777	18.556	(−26.592, 46.147)	0.610
	AP–BP	43.659	8.295	(27.401, 59.916)	<0.001

## Data Availability

Data sharing not applicable.

## Supplementary Materials

The following are available online at https://www.mdpi.com/1424-8220/21/2/560/s1, Figure S1: Romberg Test. On the x-axis, the name of each variable for each group analyzed and on the y-axis in order: Elipse in mm^2^, path, path x, path z in mm, sway area in mm^2^/s, and mean velocity, mean velocity x and mean velocity z in mm/s. Differences in the treatments effect and controls. °: Statistical significance compared to CS (*p* < 0.05). *: Statistical significance between different before and after treatments (*p* < 0.05). Figure S2: Romberg Test. Differences in the treatments effect and controls accounting for the low and high bands of the postural oscillation. Results are reported in both the medio-lateral component (x) and antero-posterior component (z). *: Statistically significant difference (*p* < 0.05) between a before-after condition. ■: Statistically significant compared to the controls (*p* < 0.05). Active: Equistasi ^®^ treatment. Figure S3: Kendall’s correlation results between sEMG and spatio-temporal parameters. (A) On the left before Equistasi, and on the right after Equistasi. (B) On the left before Placebo and on the right after Placebo. The values of the correlation coefficients are presented by the color thickness. The non-significant coefficients (*p*-value is less than 0.05) are barred. The measure unit of each spatio-temporal parameter is reported in Section 3.2. Figure S4: Kendall’s correlation results between sEMG and Romberg Test parameters before Equistasi. On the left antero posterior direction (z) during EC (top) and during EO (bottom); on the right medio-lateral direction during EC (top) and during EO (bottom). The values of the correlation coefficients are presented by the color thickness. The non-significant coefficients (*p*-value is less than 0.05) are barred. Figure S5: Kendall’s correlation results between sEMG and Romberg Test parameters after Equistasi. On the left antero posterior direction (z) during EC (top) and during EO (bottom); on the right medio-lateral direction during EC (top) and during EO (bottom). The values of the correlation coefficients are presented by the color thickness. The non-significant coefficients (*p*-value is less than 0.05) are barred. Figure S6: Kendall’s correlation results between sEMG and Romberg Test parameters before placebo. On the left antero posterior direction (z) during EC (top) and during EO (bottom); on the right medio-lateral direction during EC (top) and during EO (bottom). The values of the correlation coefficients are presented by the color thickness. The non-significant coefficients (*p*-value is less than 0.05) are barred. Figure S7: Kendall’s correlation results between sEMG and Romberg Test parameters after placebo. On the left antero posterior direction (z) during EC (top) and during EO (bottom); on the right medio-lateral direction during EC (top) and during EO (bottom). The values of the correlation coefficients are presented by the color thickness. The non-significant coefficients (*p*-value is less than 0.05) are barred.
