# Supplementary material for: Relationship between Muscular Activity and Postural Control Changes after Proprioceptive Focal Stimulation (Equistasi®) in Middle-Moderate Parkinson’s Disease Patients: An Explorative Study
Source: Sensors (Basel). 2021 Jan 14;21(2):560. doi: 10.3390/s21020560 (PMC7830724; doi:10.3390/s21020560)
Supplement: Supplementary file 1 [file sensors-21-00560-s001.pdf]

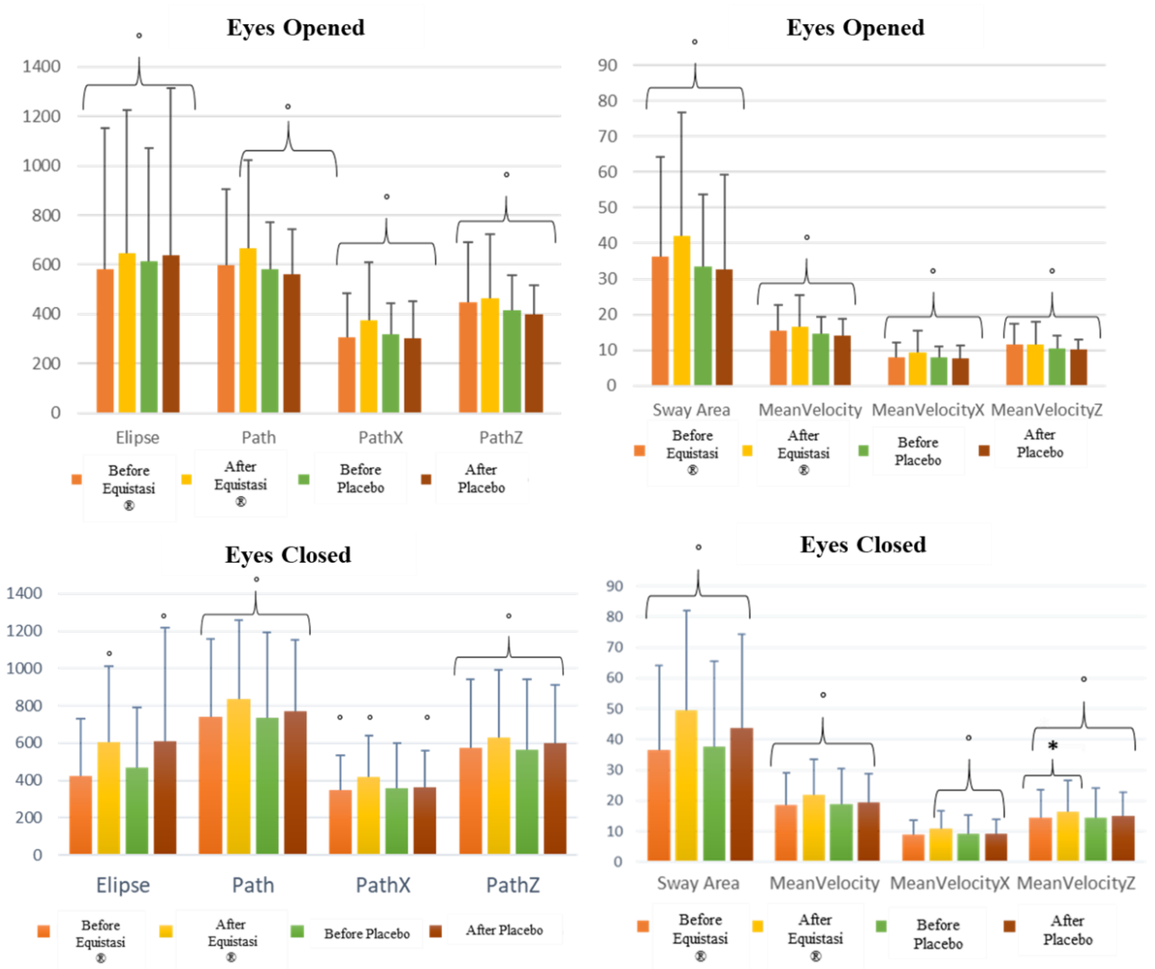

Figure S1. Romberg Test. On the x-axis, the name of each variable for each group analyzed and on the y-axis in order: Elipse in mm^2, path, path x, path z in mm, sway area in mm^2/s, and mean velocity, mean velocity x and mean velocity z in mm/s. Differences in the treatments effect and controls. °: Statistical significance compared to CS ($p < 0.05$). *: Statistical significance between different before and after treatments ($p < 0.05$).

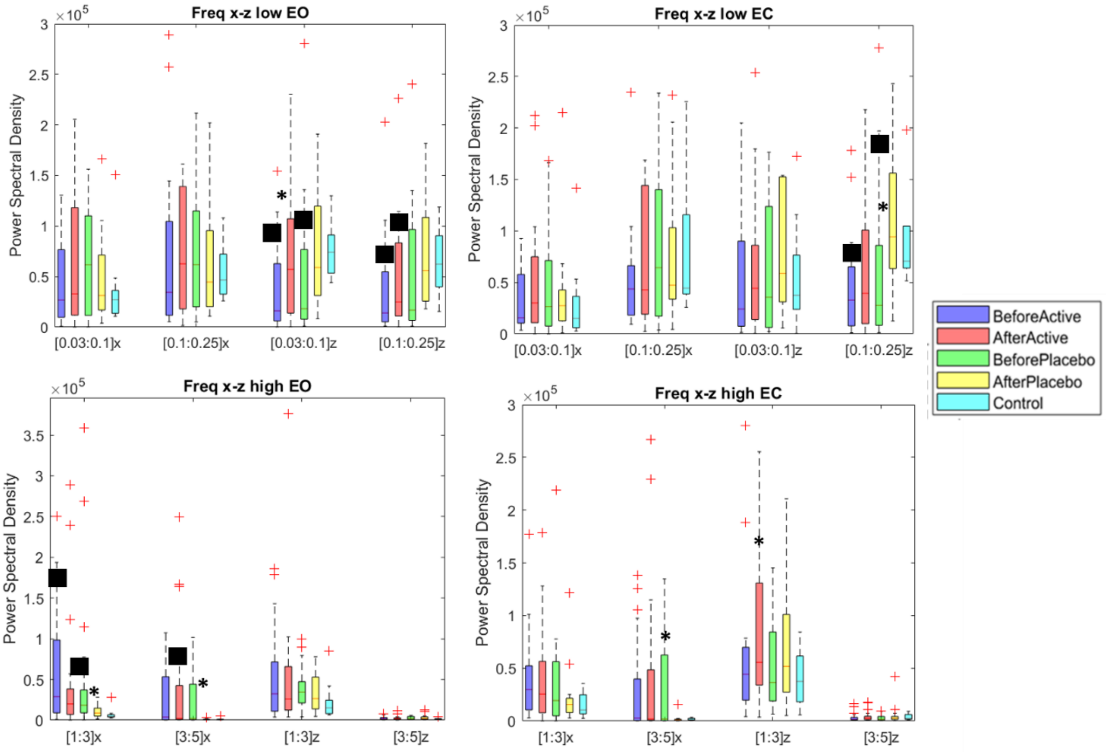

Figure S2. Romberg Test. Differences in the treatments effect and controls accounting for the low and high bands of the postural oscillation. Results are reported in both the medio-lateral component (x) and antero-posterior component (z). *: Statistically significant difference (p < 0.05) between a before-after condition. ■: Statistically significant compared to the controls (p < 0.05). Active: Equistasi ® treatment.

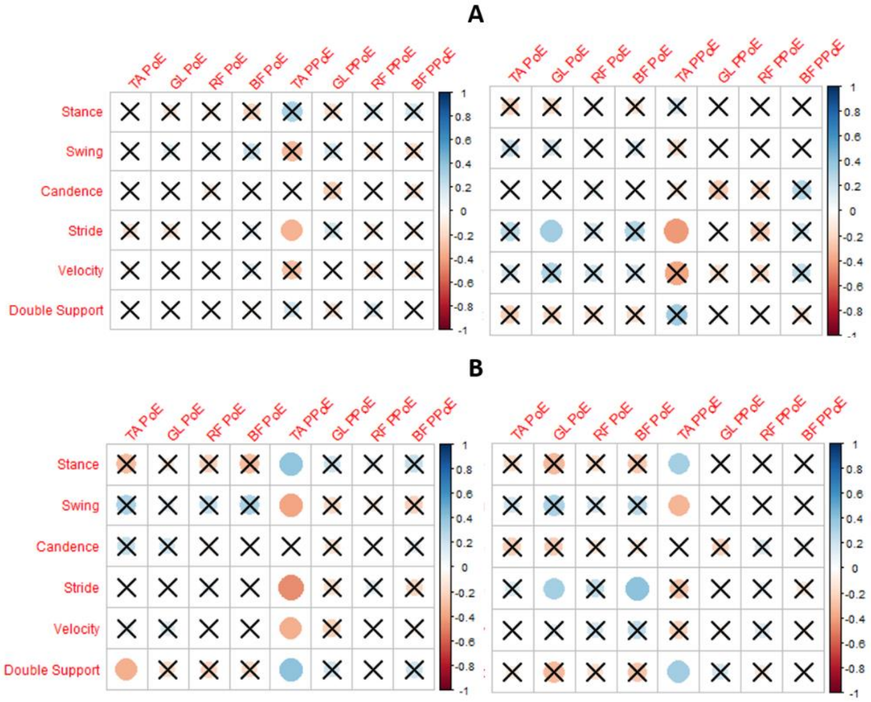

Figure S3. Kendall's correlation results between sEMG and spatio-temporal parameters. (**A**) On the left before Equistasi, and on the right after Equistasi. (**B**) On the left before Placebo and on the right after Placebo. The values of the correlation coefficients are presented by the color thickness. The non-significant coefficients (p-value is less than 0.05) are barred. The measure unit of each spatio-temporal parameter is reported in Section 3.2.

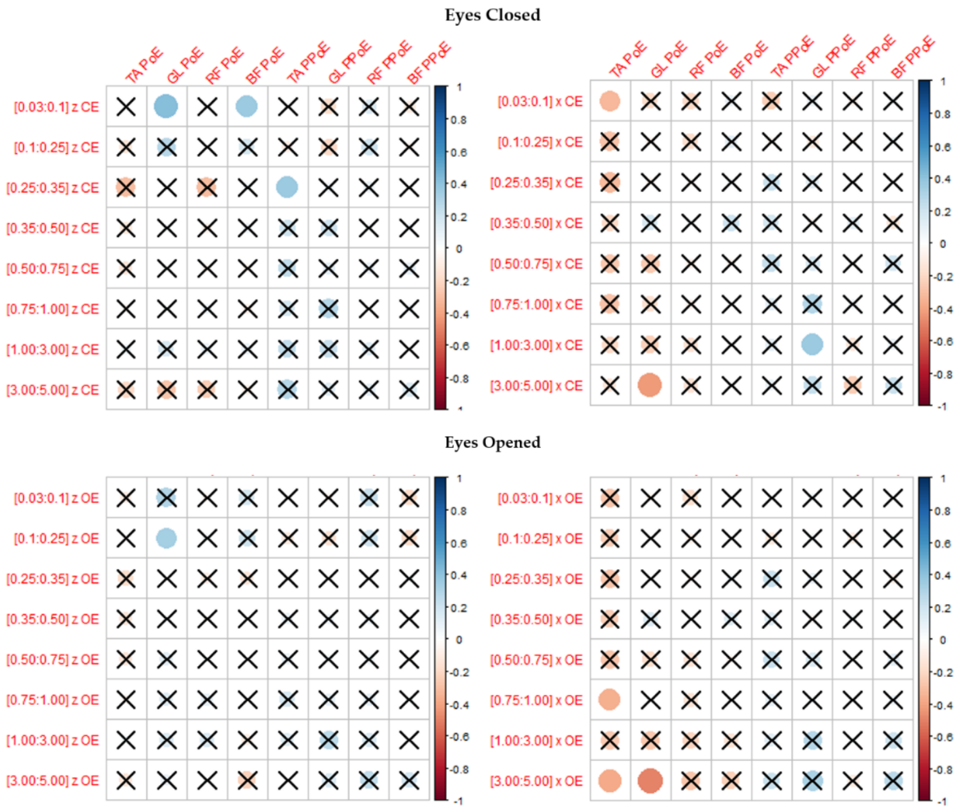

Figure S4. Kendall's correlation results between sEMG and Romberg Test parameters before Equistasi. On the left antero posterior direction (z) during EC (top) and during EO (bottom); on the right medio-lateral direction during EC (top) and during EO (bottom). The values of the correlation coefficients are presented by the color thickness. The non-significant coefficients (*p*-value is less than 0.05) are barred.

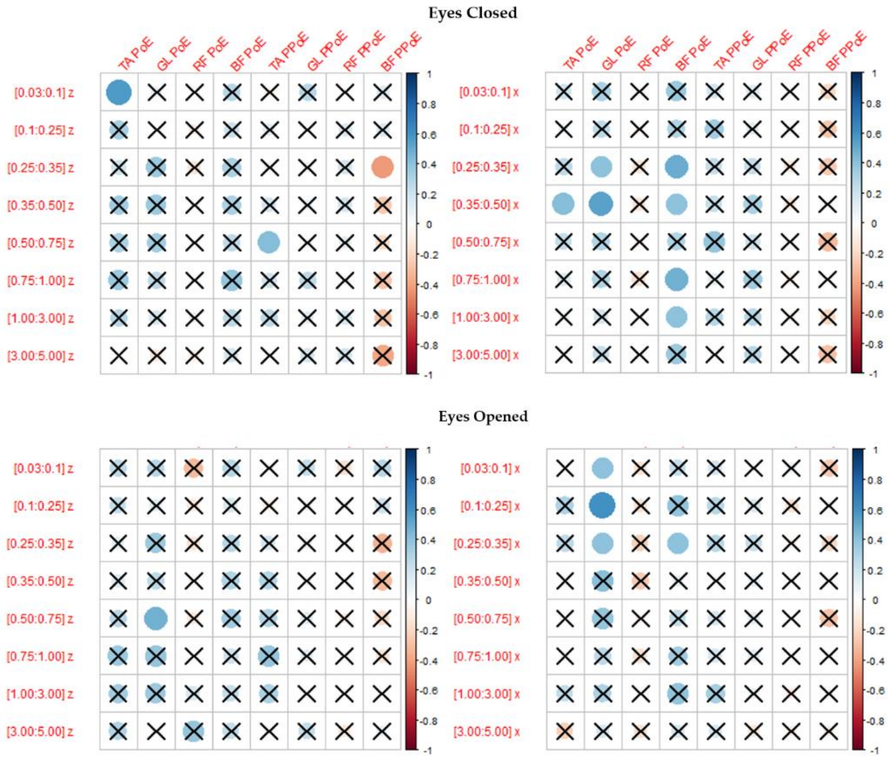

Figure S5. Kendall's correlation results between sEMG and Romberg Test parameters after Equistasi. On the left antero posterior direction (z) during EC (top) and during EO (bottom); on the right medio-lateral direction during EC (top) and during EO (bottom). The values of the correlation coefficients are presented by the color thickness. The non-significant coefficients (*p*-value is less than 0.05) are barred.

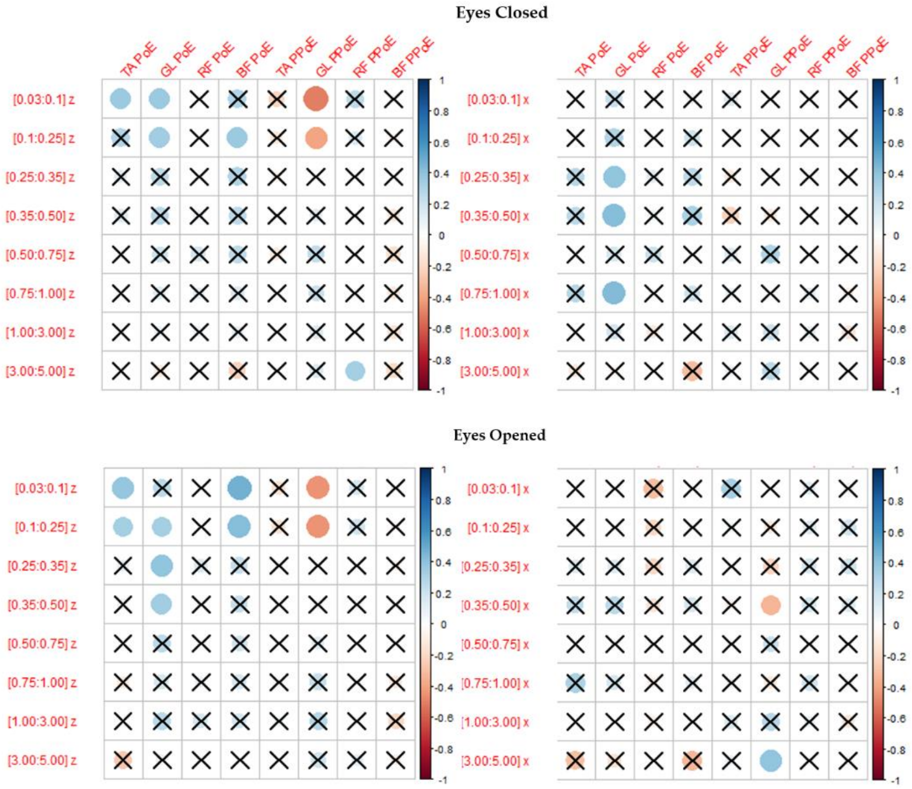

Figure S6. Kendall's correlation results between sEMG and Romberg Test parameters before placebo. On the left antero posterior direction (z) during EC (top) and during EO (bottom); on the right medio-lateral direction during EC (top) and during EO (bottom). The values of the correlation coefficients are presented by the color thickness. The non-significant coefficients (*p*-value is less than 0.05) are barred.

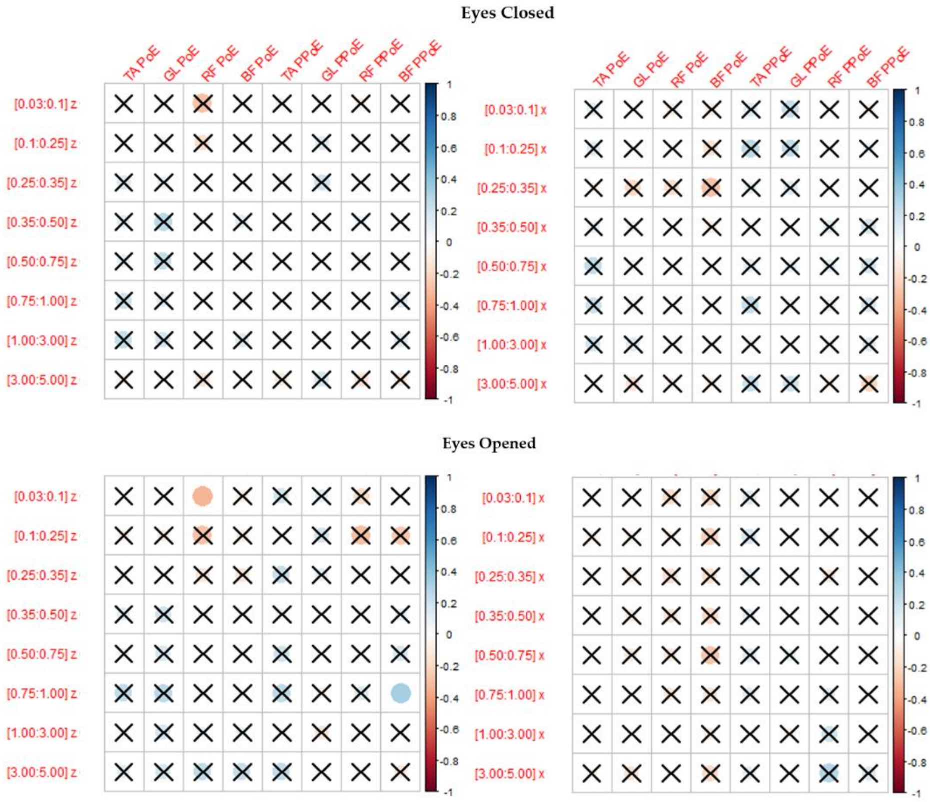

Figure S7. Kendall's correlation results between sEMG and Romberg Test parameters after placebo. On the left antero posterior direction (z) during EC (top) and during EO (bottom); on the right medio-lateral direction during EC (top) and during EO (bottom). The values of the correlation coefficients are presented by the color thickness. The non-significant coefficients (*p*-value is less than 0.05) are barred.
